# Diabetes Mellitus and Risk of Age-Related Macular Degeneration: A Systematic Review and Meta-Analysis

**DOI:** 10.1371/journal.pone.0108196

**Published:** 2014-09-19

**Authors:** Xue Chen, Shi Song Rong, Qihua Xu, Fang Yao Tang, Yuan Liu, Hong Gu, Pancy O. S. Tam, Li Jia Chen, Mårten E. Brelén, Chi Pui Pang, Chen Zhao

**Affiliations:** 1 Department of Ophthalmology, The First Affiliated Hospital of Nanjing Medical University and State Key Laboratory of Reproductive Medicine, Nanjing Medical University, Nanjing, China; 2 Department of Ophthalmology & Visual Sciences, The Chinese University of Hong Kong, Hong Kong, China; 3 Department of Ophthalmology, The Affiliated Jiangyin Hospital of Southeast University Medical College, Jiangyin, China; 4 Department of Ophthalmology, Ningbo Medical Treatment Center Lihuili Hospital, Ningbo, China; Purdue University, United States of America

## Abstract

Age-related macular degeneration (AMD) is a major cause of severe vision loss in elderly people. Diabetes mellitus is a common endocrine disorder with serious consequences, and diabetic retinopathy (DR) is the main ophthalmic complication. DR and AMD are different diseases and we seek to explore the relationship between diabetes and AMD. MEDLINE, EMBASE, and the Cochrane Library were searched for potentially eligible studies. Studies based on longitudinal cohort, cross-sectional, and case-control associations, reporting evaluation data of diabetes as an independent factor for AMD were included. Reports of relative risks (RRs), hazard ratios (HRs), odds ratio (ORs), or evaluation data of diabetes as an independent factor for AMD were included. Review Manager and STATA were used for the meta-analysis. Twenty four articles involving 27 study populations were included for meta-analysis. In 7 cohort studies, diabetes was shown to be a risk factor for AMD (OR, 1.05; 95% CI, 1.00–1.14). Results of 9 cross-sectional studies revealed consistent association of diabetes with AMD (OR, 1.21; 95% CI, 1.00–1.45), especially for late AMD (OR, 1.48; 95% CI, 1.44–1.51). Similar association was also detected for AMD (OR, 1.29; 95% CI, 1.13–1.49) and late AMD (OR, 1.16; 95% CI, 1.11–1.21) in 11 case-control studies. The pooled ORs for risk of neovascular AMD (nAMD) were 1.10 (95% CI, 0.96–1.26), 1.48 (95% CI, 1.44–1.51), and 1.15 (95% CI, 1.11–1.21) from cohort, cross-sectional and case-control studies, respectively. No obvious divergence existed among different ethnic groups. Therefore, we find diabetes a risk factor for AMD, stronger for late AMD than earlier stages. However, most of the included studies only adjusted for age and sex; we thus cannot rule out confounding as a potential explanation for the association. More well-designed prospective cohort studies are still warranted to further examine the association.

## Background

Age-related macular degeneration (AMD) has become a major cause of irreversible visual impairments in elderly people around the world, casting a heavy socio-economic burden on eye care [1], [2], [3]. AMD can be classified into the early and late stages. Patients with early AMD are usually asymptomatic, while severe vision loss frequently occurs in its late stage. Late AMD can be further categorized into two main subtypes: neovascular AMD (nAMD) and geographic atrophy (GA) [3]. The estimated prevalence is 6.8% for early AMD and 1.5% for late AMD in Caucasians over the age of 40 years [3]. It is estimated that 5% of early AMD patients will progress to late AMD over a 5-year period, increasing to nearly 15% over a 15-year period [4], [5]. Similar prevalence has been identified in Asians but not in the black population [6], [7].

The pathogenesis of AMD is complicated with multiple risk factors, including age, ocular dysfunctions, systemic diseases, diet, smoking, genetic, and environmental factors [8]. As a modifiable personal factor, whether diabetes play a role in the development and progression of AMD has been vigorously studied. While several reports presented positive correlations between diabetes and AMD [9], [10], [11], [12], [13], [14], some other reports showed no such effect [15], [16]. Even inversed relationship has been reported [17]. To gain a clear insight into the relationship between AMD and diabetes, we conducted a meta-analysis to assess whether diabetes is a risk factor for AMD.

## Methods

### Eligibility Criteria for Considering Studies for This Review

Included studies were: (1) studies evaluating diabetes as an individual risk factor for AMD; (2) prospective or retrospective cohort study, or study of cross-sectional or case-control design; (3) studies using predefined criteria and procedures for diabetes diagnosis and AMD grading; and (4) relative risks (RRs), hazard ratios (HRs), and odds ratio (ORs) have been reported, or data provided that enabled calculations of these outcomes. Case reports, reviews, abstracts, conference proceedings, editorials, reports with incomplete data, and non-English articles were excluded. For serial publications from the same research team using overlapped subjects, we included those: (1) with the latest follow-up information; and (2) providing adjusted RRs, HRs, or ORs with 95% CIs. To come up with a more precise insight into whether diabetes is an independent risk factor for AMD, only studies investigating diabetes as the main exposure, or provides adjusted RRs, HRs, or ORs with 95% CIs were included. This study was approved and reviewed by the institutional ethics committee of The First Affiliated Hospital of Nanjing Medical University and adhered to the tenets of the Declaration of Helsinki.

### Search Methods for Identifying Studies

We searched MEDLINE, EMBASE, and the Cochrane Library for all relevant articles starting from year 1946 to March 18, 2014. We followed the Cochrane Handbook for Systematic Reviews of Interventions [18] and Meta-analysis of Observational Studies in Epidemiology (MOOSE) guideline [19] in designing and reporting the current study. Our search strategies were detailed in **Appendix S1**. No language filters was applied. Additional studies were identified from reference lists of the retrieved reports. Retrieved records and eligibility status were managed using EndNote X5 software (http://endnote.com/).

### Study Selection

Two investigators (X.C. and S.S.R.) independently screened all retrieved citations based on title, abstract, and complete document if necessary. All relevant full-text articles were obtained and reviewed to determine the eligibility of each study. Disagreements were resolved via consensus with a senior reviewer (C.Z.).

### Data Collection and Risk of Bias Assessment

The two reviewers (X.C. and S.S.R.) extracted outcomes from each study separately with a customized datasheet. Data obtained included: first author, year of publication, title of the study (if any), duration of the study, country or region, races, study design, sample size, estimated ORs, RRs, or HRs, adjusted factors in multiple regression analysis, and clinical examinations and diagnostic criteria for AMD and diabetes. We used the Newcastle Ottawa Scale (NOS, accessed via http://www.ncbi.nlm.nih.gov/books/NBK35156/) [20] and the criteria recommended by Agency for Healthcare Research and Quality (AHRQ, accessed via http://www.ncbi.nlm.nih.gov/books/NBK35156/) [21] to evaluate the risk of biases for prospective cohorts or case-control studies, and cross-sectional studies, respectively. All data from these two reviewers were compared. Agreement among the reviewers was sought after completion of grading.

### Data Synthesis and Analysis

We assessed the association between diabetes and AMD by combining ORs from case-control and cross-sectional studies, and RRs or HRs from cohort studies. Heterogeneity between studies were tested by Cochran's Q statistic, and evaluated by the proportion of variation attributable to among-study heterogeneity, *I^2^*. Heterogeneity among studies was considered no, low, moderate, and high when *I^2^* equals to 0% to 24%, 25% to 49%, 50% to 74%, and more than 75%, respectively. If *p* for Q<0.1 or *I^2^*>50%, a random-effects model (the DerSimonian and Laird method) was used [22], otherwise we used a fixed-effects model(the Mantel-Haenszel method) [23]. Subgroup analysis was conducted by the study designs, AMD stages and clinical subtypes, and ethnic groups. The Asians were further divided into subgroups, including the East Asians (Japan, China, Taiwan, and Korea), Southeast Asians (Singapore), West Asians (Israel, Iran, and Turkey), and South Asians (Nepal, and India). As to the subgroup analysis concerning different AMD stages, we applied the widely accepted clinical classification system as described by the Age-Related Eye Disease Study Research Group [24], [25]. Briefly, early AMD was defined by the appearance of drusen and pigmentary alterations within 2 disc diameters of the fovea. Late AMD was featured by the presence of large drusen (soft and/or indistinct) together with pigmentary abnormalities, or can be generally recognized as nAMD and/or GA. Moreover, sensitivity analysis was conducted to affirm the estimated association by removing studies with poor quality or prone to introducing biases. Publication bias and small-study effects were assessed with funnel plots [26] and Egger's test [27]. All analyses were conducted with Review Manager version 5.2 (Cochrane Collaboration, Oxford, UK; http://ims.cochrane.org/revman) and STATA software (version 12.0; StataCorp LP, College Station, TX). Alpha was set to 0.05 for two-sided test.

## Results

### Literature

**Figure 1:**
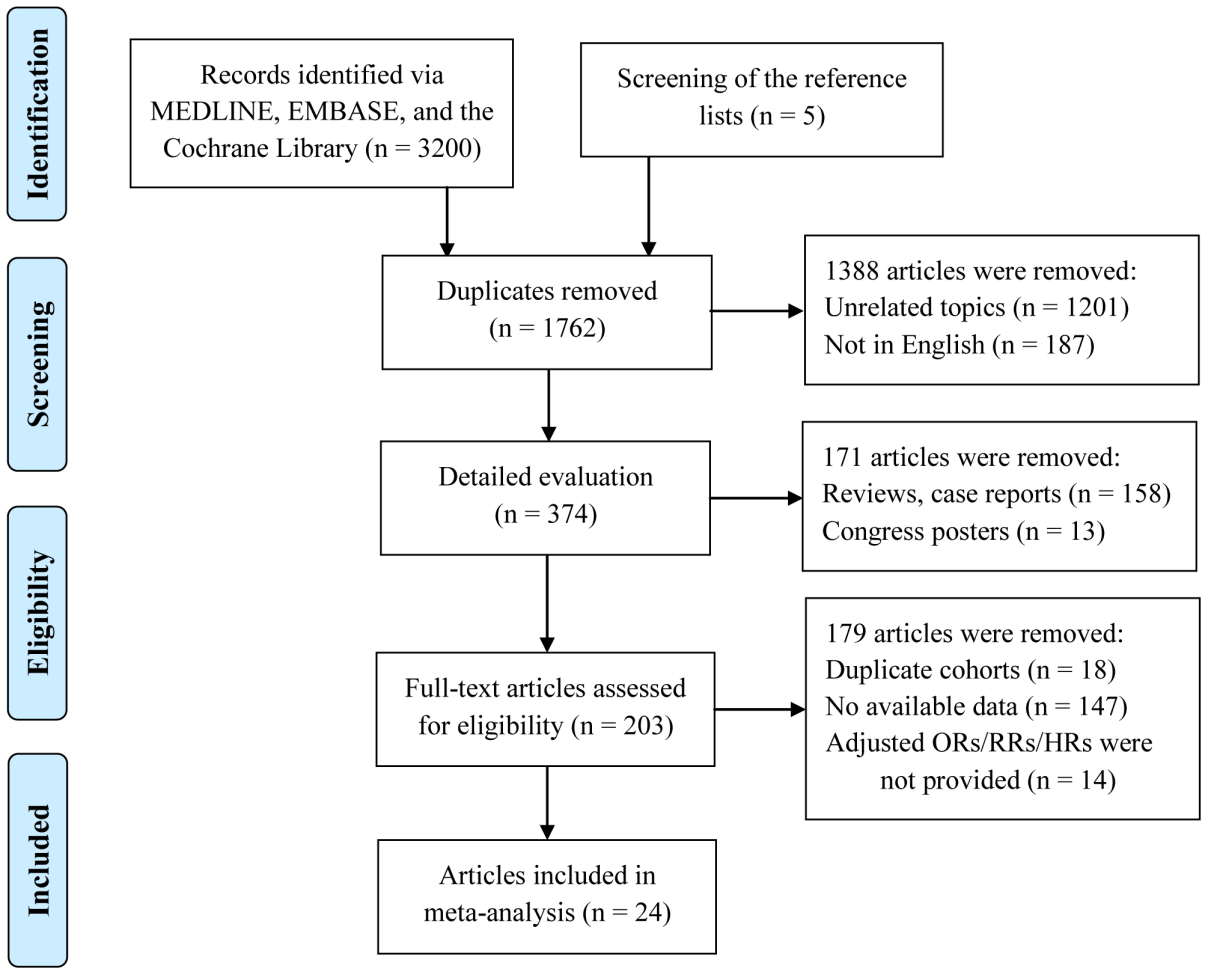
Flow chart depicting the screening process for inclusion in the meta-analysis.

A total of 3205 records were yielded from digital search and manual screen of reference list. Thirty-eight articles, published from 1986 to 2013, were included for the systematic review. Workflow of literature screen and review was shown in **Figure 1**. In addition, to provide a better understanding in diabetes as an independent risk factor for AMD, fourteen studies that presented diabetes as a covariate and provided ORs/RRs/HRs from baseline data without any adjustment were excluded, involving 12 cross-sectional [11], [15], [17], [28], [29], [30], [31], [32], [33], [34], [35], [36] and 2 case-control studies [37], [38]. The 24 articles included 1858350 participants in 27 independent study populations, comprising 7 cohort studies [39], [40], [41], [42], [43], 9 cross-sectional studies [9], [14], [16], [44], [45], [46], [47], [48], and 11 case-control studies [12], [13], [36], [49], [50], [51], [52], [53], [54], [55], [56]. Among the 27 study populations, 10 were in Asia, 9 in North America (United States), 6 in Europe, 1 in Oceania (Australia), and 1 in South America (Barbados). Most studies used predefined criteria for AMD diagnosis and adopted standard grading system [57], [58]. Samples sizes varied widely, from less than 50 to over 1.5 million (**Table 1**). Only two of the earliest studies, in 1986 [49] and 1998 [50], had sample sizes less than 100. Risk of bias assessments for cohort, cross-sectional, and case-control studies has been performed (**Tables S1–S3**). Tan et al [59] and Tomany et al [42] both involved the Blue Mountain Eye Study cohort, we included latter one in the analysis. The ORs/RRs/HRs with 95% CI and the corresponding adjusted variables for each study were listed in overall AMD, early AMD or late AMD (**Table 2**).

**Table 1:** Characteristics of Included Cohorts.

First Author (Publication Year)	Study	Study Period	Region	Race	Sample Size	Diagnostic Criteria	
						AMD	Diabetes
***Cohort Studies (Prospective & Retrospective)***							
Tomany et al (2004)	BDES	1993–1995	US	Caucasian	3562	I & W	PGL & S
	BMES	1997–1999	Australia	Caucasian	2330	I & W	PGL & S
	RS	1997–1999	Netherlands	Caucasian	3631	I & W	PGL & S
Leske et al (2006)	BISED II	1987–1992	Barbados	Mixed	2793	W	M & S
Yasuda et al (2009)	Hisayama Study	2007	Japan	East Asian	1401	I & W	M & PGL
Shalev et al (2011)	MHS	1998–2007	Israel	West Asian	108973	ICD9	M
Hahn et al (2013)	NA	1995–2005	US	Caucasian	16510	ICD9	ICD9
***Cross-sectional Studies***							
Delcourt et al (2001)	POLA	1995–1997	France	Caucasian	2584	I & W	Interview
Vaičaitienė et al (2003)	NA	1995–1997	Lithuania	Caucasian	438	NA	NA
Duan et al (2007)	NA	2000–2001	US	Caucasian	1519086	ICD9	ICD9
Klein et al (2007)	WHISE	1993–2002	US	Caucasian	4288	W	M
Topouzis et al (2009)	EUREYE study	2000–2003	Europe¶	Caucasian	4722	I	S
Xu et al (2009)	Beijing Eye Study	2006	China	East Asian	2960	W	PGL & S
Choi et al (2011)	NA	2006–2008	Korea	East Asian	3008	W	M & PGL
Cheung et al (2013)	SIES	2007–2009	Singapore	Southeast Asian	3337	W	PGL & S
	CIEMS	2006–2008	India	South Asian	3422	W	PGL & S
***Case-control Studies***							
Blumenkranz et al (1986)	NA	NA	US	Caucasian	49	NA	PGL
Ross et al (1998)	NA	NA	US	Caucasian	94	Detailed in paper.	M
McGwin et al (2003)	NA	1997–2001	US	Caucasian	6050	ICD9	ICD9
Moeini et al (2005)	NA	2001	Iran	West Asian	130	NA	PGL
Alexander et al (2007)	NA	2001–2003	US	Caucasian	62179	ICD9	ICD9
Kim et al (2008)	NA	1998–2003	US	Caucasian	204	W	Questionnaire
Lin et al (2008)	NA	2002–2006	Taiwan	East Asian	280	I	NA
Nitsch et al (2008)	GPRD	1987–2002	UK	Caucasian	104176	Readcodes & OXMIS	M
Cackett et al (2011)	NA	2007–2008	Singapore	Southeast Asian	1617	W	Questionnaire
Sogut et al (2013)	NA	NA	Turkey	West Asian	280	W	ADA
Torre et al (2013)	NA	2011	Italy	Caucasian	246	NA	Questionnaire

¶ Europe: Estonia, France, Greece, Italy, Norway, Spain, UK;

Abbreviation: BDES: Beaver Dam Eye Study; BMES: Blue Mountains Eye Study; RS: Rotterdam Study; BISED II: Barbados Incidence Study of Eye Diseases; MHS: Maccabi Healthcare Services; NA: not available; POLA: Pathologies Oculaires Liées àl'Age Study; WHISE: Women's Health Initiative Sight Examination; SIES: Singapore Indian Eye Study; CIEMS: Central India Eye and Medical Study; GPRD: General Practice Research Database; AMD: Age Related Macular Degeneration; &: represents a combination of two diagnostic methods; I: International Classification and Grading System for AMD; W: Wisconsin Age-Related Maculopathy Grading System; ICD9: International Classification of Diseases with Clinical Modifications, Ninth Revision; PGL: Plasma Glucose Level; S: Self-reported diabetic history or medications; M: Medical recorded diabetic history or medications; ADA: American Diabetes Association diagnostic criteria.

**Table 2:** Detailed Analytical Information for Included Cohorts.

First Author(Publication Year)	OR/RR/HRΔ [95% CI]					Adjusted Variables
	*Early AMD*	*Late AMD*			*AMD*	
		*nAMD*	*GA*	*Total*		
***Cohort Studies (Prospective & Retrospective)***						
Tomany (2004)	—	0.67 [0.24, 1.86]	2.05 [0.84, 4.99]	1.21 [0.62, 2.36]	1.21 [0.62, 2.36]	Age, Sex
*BDES*	—	—	0.79 [0.10, 6.31]	—	—	Age, Sex
*BMES*	—	—	8.31 [2.34, 29.50]	—	—	Age, Sex
*RS*	—	—	0.79 [0.10, 6.19]	—	—	Age, Sex
Leske (2006)	0.88 [0.60, 1.30]	—	—	2.70 [1.00, 7.30]	1.02 [0.71, 1.47]	Age
Yasuda (2009)	0.70 [0.37, 1.31]	—	—	0.69 [0.16, 2.95]	0.69 [0.39, 1.24]	Multiple Factors#
Shalev (2011)	—	—	—	—	1.18 [1.01, 1.38]	Mutually adjusted
Hahn (2013)	—	1.11 [0.97, 1.27]	1.03 [0.97, 1.09]	1.04 [0.99, 1.10]	1.04 [0.99, 1.10]	Multiple Factors*
***Cross-sectional Studies***						
Delcourt (2001)	—	—	—	1.22 [0.45, 3.29]	1.22 [0.45, 3.29]	Age, Sex
Vaičaitienė (2003)	—	—	—	—	4.61 [2.45, 8.67]	Age, Sex
Duan (2007)	—	1.48 [1.44, 1.51]	—	1.48 [1.44, 1.51]	1.18 [1.16, 1.19]	Age, Sex, Race
Klein (2007)	0.87 [0.67, 1.12]	2.49 [1.17, 5.31]	2.28 [0.63, 8.28]	2.43 [1.26, 4.70]	0.94 [0.74, 1.20]	Age
Topouzis (2009)	0.98 [0.83, 1.17]	1.81 [1.10, 2.98]	1.06 [0.28, 4.04]	1.38 [0.90, 2.12]	1.01 [0.85, 1.19]	Multiple Factors‡
Xu (2009)	1.30 [0.69, 2.43]	—	—	1.13 [0.14, 9.40]	1.28 [0.70, 2.34]	None
Choi (2011)	1.87 [1.07, 3.28]	—	—	—	1.87 [1.07, 3.28]	Multiple Factors†
Cheung (2013)						
*SIES*	0.93 [0.68, 1.28]	—	—	—	0.93 [0.68, 1.28]	Age, Sex
*CIEMS*	1.14 [0.47, 2.77]	—	—	—	1.14 [0.47, 2.77]	Age, Sex
***Case-control Studies***						
Blumenkranz (1986)	—	—	—	0.53 [0.06, 4.71]	0.53 [0.06, 4.71]	Use siblings
Ross (1998)	—	—	—	—	1.09 [0.21, 5.59]	Age
McGwin Jr (2003)	—	—	—	—	1.78 [1.43, 2.20]	Age, Sex
Moeini (2005)	—	—	—	—	1.29 [0.52, 3.21]	Age, Sex, Risk factors
Alexander (2007)	—	1.16 [1.11, 1.21]	—	1.16 [1.11, 1.21]	1.16 [1.11, 1.21]	Age, Sex, Race, Database length
Kim (2008)	—	0.61 [0.27, 1.39]	—	0.61 [0.27, 1.39]	0.61 [0.27, 1.39]	Use siblings
Lin (2008)	—	1.20 [0.44, 3.26]	0.97 [0.36, 2.63]	1.07 [0.45, 2.57]	1.07 [0.45, 2.57]	Age, Sex
Nitsch (2008)	—	—	—	—	1.36 [1.29, 1.43]	Age, Sex, Practice, Consultation Rate
Cackett (2011)	—	0.92 [0.50, 1.70]	—	0.92 [0.50, 1.70]	0.92 [0.50, 1.70]	Age, Sex
Sogut (2013)	—	—	—	1.68 [0.76, 3.69]	1.68 [0.76, 3.69]	Age, Sex
Torre (2013)	—	—	—	—	0.80 [0.08, 8.07]	Age, Sex, Smoking

Δ OR is for cross-sectional and case-control studies, RR is for prospective cohort studies, HR is for retrospective cohort studies;

# Age, Sex, Smoking habit, White blood cells;

*Age, Sex, Race, History of hypertension, Atherosclerosis, Stroke, Coronary heart disease, Hyperlipidemia, Charlson index;

‡ Age, Sex, Smoking, Education, BMI, Alcohol consumption, Cardiovascular disease, Aspirin use, Systolic blood pressure, Alpha-tocopherol ratio, Vitamin C, Lutein;

† Age, Sex, Current smoking, Obesity, Hypertension.

Abbreviations: OR: odds ratio; RR: risk ratio; HR: hazard ratio; CI: confidence interval; AMD: Age-related macular degeneration; nAMD: neovascular AMD; GA: geographic atrophy; WBC: white blood cell.

### Meta-Analysis

**Figure 2:**
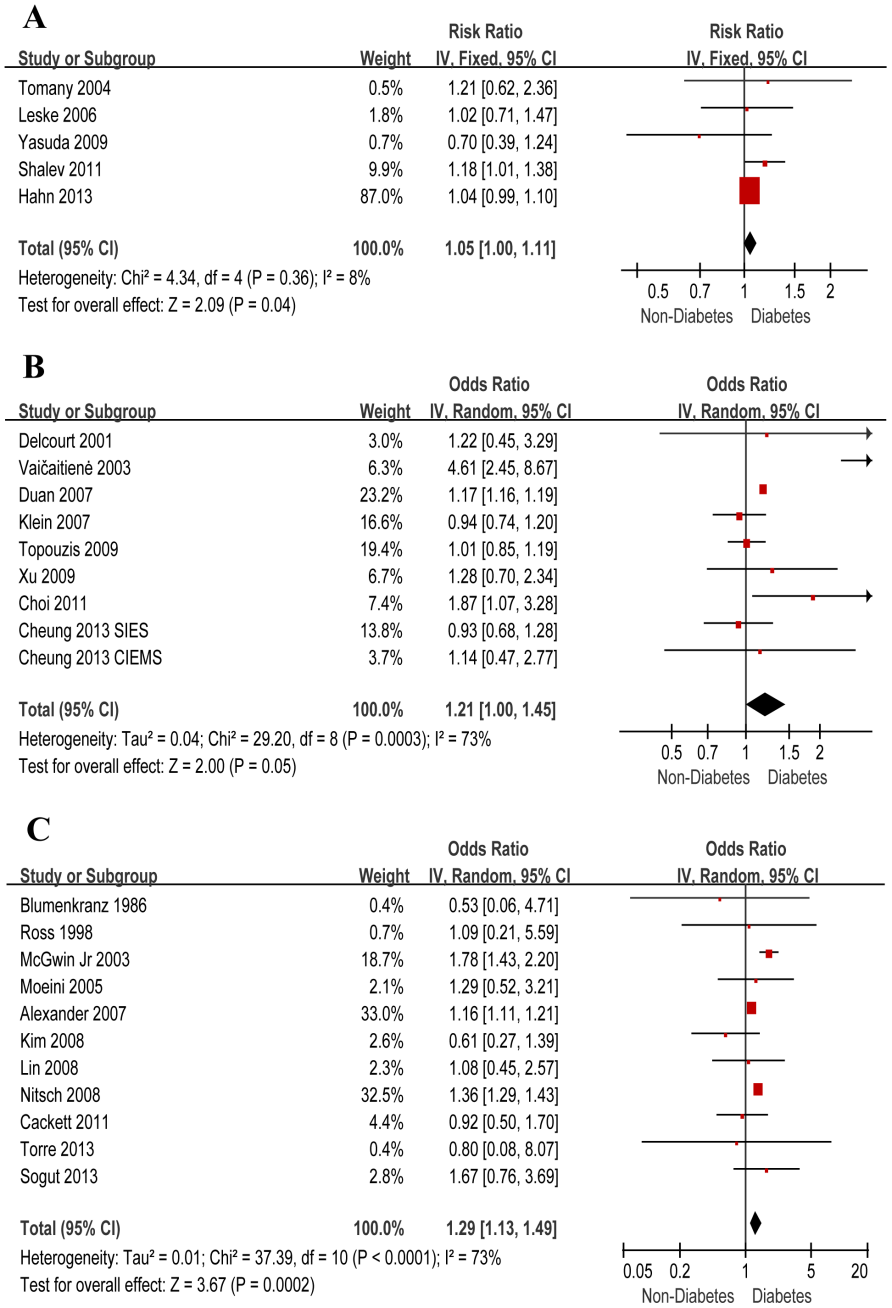
Effects of diabetes on AMD risks. Graphs showing the effects of diabetes on the risk of Age-related Macular Degenerations in longitudinal cohort studies (A), cross-sectional studies (B), and case-control studies (C). IV: inverse variance, CI: confidence interval.

The effects of diabetes on the risk of AMD in all these studies were found to be essentially consistent (**Figure 2** **and** **Table 3**). According to the meta-analysis of 7 cohort studies, diabetes was associated with AMD (OR, 1.05; 95% CI, 1.00–1.11). Subgroup analysis based on AMD stages revealed diabetes as a marginal risk factor for late AMD (OR, 1.05; 95% CI, 0.99–1.10), but not for its early form (OR, 0.83; 95% CI, 0.60–1.15). Subgroup analysis by AMD subtypes showed that the pooled OR of diabetes for risk of nAMD was 1.10 (95% CI, 0.96–1.26), for risk of GA was 1.63 (95% CI, 0.51–5.21). In the 9 cross-sectional study populations, diabetes was found increasing AMD risk (OR, 1.21; 95% CI, 1.00–1.45). Subgroup analysis confirmed this effect for late AMD (OR, 1.48; 95% CI, 1.44–1.51), and nAMD (OR, 1.48; 95% CI, 1.44–1.51), but not for early AMD (OR, 0.99; 95% CI, 0.88–1.12) or GA (OR, 1.58; 95% CI, 0.63–3.99). The results kept consistent in the analysis of 11 case-control studies. The pooled OR of diabetes for AMD was 1.29 (95% CI, 1.13–1.49). The pooled OR was 1.16 (95% CI, 1.11–1.21) for late AMD, and 1.15 (95% CI, 1.11–1.21) for nAMD. To reduce the methodological heterogeneity and the potential effect led by other risk factors, we also conducted meta-analysis solely using multivariate-adjusted outcomes. Only 3 cohort studies and 2 cross-sectional were included, and the results varied from the overall data, which was probably due to the limited number of included studies. However, in both groups, diabetes was found as a marginal risk factor for nAMD in cross-sectional studies (OR, 1.04; 95% CI, 0.99–1.10) and a solid risk factor for late AMD in cohort studies (OR, 1.81; 95% CI, 1.10–2.98). No association between diabetes and early AMD or GA was found in both groups (**Table 4**). Subgroup analyses by ethnic group were further performed. The associations of diabetes and overall and early AMD were similar between the Asian and Caucasian populations (**Table 5**), while associations between diabetes and all subtypes of late AMD were suggested only for the Caucasian group, but not for the overall Asian population or any of its subgroups. No indication of any obvious asymmetry was observed according to the shapes of Begg's funnel plots and Egger's test for all groups as detailed in **Tables 3** **–** **5**.

**Table 3:** Analysis of Diabetes as a Risk Factor for AMD in Different AMD Types.

Study Design	No. of Cohorts	Sample Size	Overall Effect			Heterogeneity		*Egger*'*s Test*
			*OR/RR* * *[95% CI]*	*Z score*	*p value*	*I^2^ (%)*	*Q (p)*	
***Cohort Studies***								
AMD	7	139200	1.05 [1.00, 1.11]	2.09	0.037	8	0.361	0.961
Early AMD	2	4194	0.83 [0.60, 1.15]	1.12	0.261	0	0.529	NA
Late AMD	6	30227	1.05 [0.99, 1.10]	1.70	0.088	25	0.260	0.504
nAMD	4	26033	1.10 [0.96, 1.26]	1.40	0.160	0	0.335	NA
GA	4	26033	1.63 [0.51, 5.21]†	0.83	0.407	72	0.014	0.523
***Cross-sectional Studies***								
AMD	9	1543845	1.21 [1.00, 1.45]†	2.00	0.045	73	0.000	0.813
Early AMD	6	21737	0.99 [0.88, 1.12]	0.15	0.883	28	0.224	0.205
Late AMD	5	1533640	1.48 [1.44, 1.51]	32.20	0.000	0	0.642	0.774
nAMD	3	1528096	1.48 [1.44, 1.51]	32.23	0.000	20	0.287	0.154
GA	2	9010	1.58 [0.63, 3.99]	0.97	0.333	0	0.419	NA
***Case-control Studies***								
AMD	11	175305	1.29 [1.13, 1.49]†	3.67	0.000	73	0.000	0.976
Late AMD	6	64609	1.16 [1.11, 1.21]	6.65	0.000	0	0.520	0.334
nAMD	4	62179	1.15 [1.11, 1.21]	6.55	0.000	0	0.416	0.257
GA	1	280	0.97 [0.36, 2.63]	0.06	0.954	NA	NA	NA

* OR is for cross-sectional and case-control studies, RR is for cohort studies.

† Studies using random effect model.

Abbreviations: OR: odds ratio; RR: risk ratio; CI: confidence interval; AMD: age related macular degeneration; nAMD: neovascular AMD; GA: geographic atrophy; NA: not available.

**Table 4:** Analysis of Diabetes as a Risk Factor for AMD in Different AMD Types with Multivariate-adjusted ORs/RRs/HRs.

Study Design	No. of Cohorts	Sample Size	Overall Effect			Heterogeneity		Egger's Test
			*OR/RR* * *[95% CI]*	*Z score*	*p value*	*I^2^ (%)*	*Q (p)*	
***Cohort Studies***								
AMD	3	126884	1.07 [0.93, 1.22]†	0.96	0.339	52	0.125	0.904
Early AMD	1	1401	0.70 [0.37, 1.31]	1.12	0.262	NA	NA	NA
Late AMD	2	17911	1.04 [0.99, 1.10]	1.56	0.118	0	0.574	NA
nAMD	1	16510	1.11 [0.97, 1.27]	1.52	0.129	NA	NA	NA
GA	1	16510	1.03 [0.97, 1.09]	0.94	0.349	NA	NA	NA
***Cross-sectional Studies***								
AMD	2	7730	1.29 [0.71, 2.35]†	0.85	0.397	77	0.038	NA
Early AMD	2	7730	1.28 [0.69, 2.39]†	0.78	0.640	78	0.031	NA
Late AMD	1	4722	1.38 [0.90, 2.12]	1.46	0.145	NA	NA	NA
nAMD	1	4722	1.81 [1.10, 2.98]	2.34	0.020	NA	NA	NA
GA	1	4722	1.06 [0.28, 4.04]	0.09	0.928	NA	NA	NA

* OR is for cross-sectional and case-control studies, RR is for cohort studies.

† Studies using random effect model.

Abbreviations: OR: odds ratio; RR: risk ratio; CI: confidence interval; AMD: age related macular degeneration; nAMD: neovascular AMD; GA: geographic atrophy; NA: not available.

**Table 5:** Analysis of Diabetes as a Risk Factor for AMD in Different Ethnic Groups.

Ethnic Group	No. of cohorts	Sample Size	Overall Effect			Heterogeneity		*Egger*'*s Test*
			*OR/RR* * *[95% CI]*	*Z score*	*p value*	*I^2^ (%)*	*Q (p)*	
***AMD***								
Caucasian	16	1730149	1.20 [1.12, 1.29]†	4.86	0.000	84	0.000	0.733
Asian	10	125408	1.14 [1.01, 1.29]	2.11	0.035	2	0.423	0.978
* East Asian*	4	7649	1.18 [0.86, 1.61]	1.03	0.301	49	0.115	0.856
* West Asian*	3	109383	1.20 [1.03, 1.39]	2.35	0.019	0	0.688	0.385
* Southeast Asian*	2	4954	0.93 [0.70, 1.23]	0.50	0.616	0	0.973	NA
* South Asian*	1	3422	1.14 [0.47, 2.77]	0.29	0.771	NA	NA	NA
Total	27	1858350	1.18 [1.11, 1.26]†	5.11	0.000	74	0.000	0.909
***Early AMD***								
Caucasian	2	9010	0.95 [0.82, 1.09]	0.77	0.442	0	0.422	NA
Asian	5	14128	1.06 [0.85, 1.34]	0.54	0.588	40	0.152	0.626
* East Asian*	3	7369	1.21 [0.68, 2.14]†	0.65	0.517	62	0.070	0.385
* Southeast Asian*	1	3337	0.93 [0.68, 1.28]	0.43	0.667	NA	NA	NA
* South Asian*	1	3422	1.14 [0.47, 2.77]	0.29	0.771	NA	NA	NA
Total	8	25931	0.97 [0.86, 1.09]	0.53	0.595	16	0.303	0.478
***Late AMD***								
Caucasian	11	1619145	1.25 [1.05, 1.49]†	2.50	0.013	96	0.000	0.479
Asian	5	6538	1.09 [0.73, 1.63]	0.44	0.663	0	0.770	0.882
* East Asian*	3	4641	0.97 [0.48, 1.97]	0.07	0.941	0	0.865	0.800
* Southeast Asian*	1	1617	0.92 [0.50, 1.70]	0.26	0.795	NA	NA	NA
* West Asian*	1	280	1.68 [0.76, 3.69]	1.28	0.200	NA	NA	NA
Total	17	1628476	1.25 [1.07, 1.46]†	2.74	0.006	92	0.000	0.454
***Neovascular AMD***								
Caucasian	9	1616512	1.29 [1.09, 1.54]†	2.92	0.003	94	<0.001	0.524
Asian	2	1897	0.99 [0.59, 1.67]	0.04	0.970	0	0.662	NA
* East Asian*	1	280	1.20 [0.44, 3.26]	0.35	0.724	NA	NA	NA
* Southeast Asian*	1	1617	0.92 [0.50, 1.70]	0.26	0.795	NA	NA	NA
Total	11	1618409	1.27 [1.07, 1.50]†	2.81	0.005	93	<0.001	0.429
***Geographic Atrophy***								
Caucasian	6	24408	1.97 [1.07, 3.64]	2.17	0.030	35	0.172	0.178
Asian	1	280	0.97 [0.36, 2.63]	0.06	0.954	NA	NA	NA
*East Asian*	1	280	0.97 [0.36, 2.63]	0.06	0.954	NA	NA	NA
Total	7	24688	1.62 [0.96, 2.74]	1.82	0.069	34	0.166	0.687

* OR is for cross-sectional and case-control studies, RR is for prospective cohort studies.

† Studies using random effect model.

Abbreviations: OR: odds ratio; RR: relative risk; CI: confidence interval; AMD: age related macular degeneration.

### Risk of Bias Assessment and Sensitivity Analysis

In our assessment, we found most studies have a robust design and reported in a clear manner, thus have lower risks in introducing bias (**Tables S1–S3**). However, we did identify one cross-sectional study which had relative higher risk to introduce biases when used to evaluate risk-modifying effect of diabetes for AMD [14] (**Tables S2**), thus were subjected to sensitivity analysis. In sensitivity analysis, we sequentially omitted one study at a time and removed studies of higher risk of introducing bias to affirm the associations. Sensitivity analyses revealed that the study conducted by Alexander et al [52] contributed to the heterogeneity in the subgroup analysis of case-control studies, but did not alter the results in each subgroup. When removing the studies conducted by Shalev et al and Hahn et al in the subgroup analysis of cohort studies, respectively, although the *p* values for diabetes and AMD became insignificant, the direction of ORs was kept and associations of marginal significance were revealed (removing study by Shalev et al: OR, 1.04; 95% CI, 0.99–1.10; Hahn et al: OR, 1.12; 95% CI, 0.98–1.29). Similar findings were revealed by subgroup analyses involving cross-sectional and case-control studies. In the analysis of cross-sectional studies, the removal of studies by Vaičaitienė et al [14], Duan et al [45], Xu et al [16], and Choi et al [47] would also lead to borderline results (removing study by Vaičaitienė et al: OR, 1.10; 95% CI, 0.98–1.23; Duan et al: OR, 1.30; 95% CI, 0.97–1.73; Xu et al: OR, 1.21; 95% CI, 0.99–1.47; Choi et al: OR, 1.16; 95% CI, 0.96–1.41). In addition, in the subgroup analysis of case-control studies, an association of borderline significance between diabetes and AMD (OR, 1.23; 95% CI, 0.97–1.56) was presented if the study by Nitsch et al [13] was excluded.

## Discussion

Diabetes is a major concern in ophthalmic care. Whether it contributes to the prevalence of AMD has been an unsolved dilemma targeted by a large number of studies. However, obvious inconsistencies between studies, including a few large cohorts, suggest the necessity to conduct an exhaustive review and quantitative analysis on all the evidences to determine its effect. In the present systemic review and meta-analysis, we reviewed 3205 published reports and completed analysis on 1858350 participants of 27 study populations from 24 original studies. We found that diabetes is a risk factor for AMD, especially for nAMD. To our knowledge, this is the first meta-analysis addressing the topic for AMD and all its subtypes, and by using data from a comprehensive collection of prospective and retrospective cohort, cross-sectional, and case-control studies.

Clinically, AMD can be classified based on drusen features and retinal pigment epithelial abnormalities, we found most included studies follow the Wisconsin Age-related Maculopathy Grading Scheme, according to 4 levels: level 1 (no AMD), level 2 and 3 (early AMD), and level 4 (late AMD) [57], [60]. The contribution of diabetes to early AMD is inconsistent in studies. Diabetic patients have increased occurrence of early AMD in a cross-sectional study of a Korean cohort of 3008 adults [47]. No similar association has been observed in other studies. An inverse relationship is observed in the Age-Related Eye Disease Study (AREDS) [17]. In the Beaver Dam Eye Study (BDES), diabetes was found to be a protective factor for incident reticular drusen based on a 15-year cumulative incidence [61]. In this meta-analysis, no clear association was detected between diabetes and early AMD based on 16 relevant cohorts.

The associations of diabetes with late AMD are also inconsistent among previous reports. According to analysis from 5 cross-sectional and 6 case-control studies, diabetes is significantly correlated with late AMD, especially with nAMD, but not for GA. Temporal relationships revealed by 7 cohort studies further supports diabetes as a potential risk factor for late AMD, only for nAMD but not for GA. However, an association between diabetes and GA was identified in Caucasians. Also, the Blue Mountains Eye Study (BMES) has revealed diabetes as a predictor of incident GA, but not incident nAMD. This is consistent with a cross-sectional baseline report [62], [63], to 5-year [42] and 10-year [59] incident reports, providing evidence for a diabetes and GA association.

In the current meta-analysis, we found no obvious ethnic divergence regarding the association between the diabetes and risk of overall AMD and its early form. The results obtained from different Asian groups are consistent in all types of AMD. However, the association between diabetes and late AMD in the Caucasian population differs from that in the Asian population, which is probably due to the large variation of genetic factors among different ethnic groups [64], and the differences in dietary habits and lifestyles.

The biological interplay between diabetes and AMD is complicated and has not been fully elucidated. First, diabetic conditions may lead to the accumulation of the highly stable advanced glycation end products (AGEs) in multiple tissues, including the retinal pigment epithelium (RPE) cell layers and photoreceptors [51], [65]. These AGEs would first contribute to the modification of molecules, leading to the activation of NFκB, NFκB nuclear translocation, and up-regulation in the expression of the receptor for AGEs (RAGE) [66]. Further, the up-regulated RAGE, which usually localized to the neuroglia in the inner retina [67], would integrate with AGE, thus leading to high levels of the nondegradable aggregates AGE-RAGE ligands in retina [65]. Therefore, accumulated AGEs would reduce the dosage dependent RAGE-mediated activation of RPE/photoreceptor cells [68]. AGEs and RAGE were found in the RPE or both RPE and photoreceptors in the maculas of human donor retina from patients with AMD, but not in normal eyes [66], [68], indicating that AGE deposition and RAGE up-regulation in diabetic conditions are implicated in the pathogenesis of AMD.

Second, hyperglycemia and dyslipidemia in diabetic patients will disturb homeostasis of the retina by inducing inflammatory responses in tissue cells, including oxidative stress [69]. Significantly elevated oxidative stress markers and total oxidative stress (TOS), as well as decreased total anti-oxidant capacity (TAC), are found in the serum of AMD patients when compared with age-matched controls free of AMD [70], [71]. Meanwhile, anti-oxidants and omega-3 fatty acids have been shown to help with the preservation of RPE health and prevent retinal degeneration in animal models [72], [73]. Therefore, oxidative stress is recognized as one of the principle pathogenic elements in AMD [74]. Oxidative stress may further activate NF-κB regulated inflammatory genes and lead to inflammation, which would in turn generate reactive oxygen species and aggregate oxidative stress. Inflammation disrupts the NF-κB, JUN N-terminal kinase (JNK), and the NADPH oxidase pathways, consequently dysregulations of many inflammatory cytokines and chemokines, involving the tumor necrosis factor (TNF), interleukin-6 (IL-6), IL-1β, C-reactive protein (CRP), CC-chemokine ligand 2 (CCL2), and adipokines [75]. These inflammatory activations would lead to the dysfunction and even death of the RPE/photoreceptor cells [69]. Thus, oxidative stress and inflammations in the retina are pre-requites for development of AMD [74].

Meanwhile, diabetic microangiopathy shares common pathogenic pathways with AMD. Hyperglycemia and dyslipidemia in diabetic patients will lead to multiple microvascular complications, including diabetic retinopathy (DR). AMD and DR share some common features in pathogenesis and treatment. In a longitudinal study over 10 years, individuals with DR, including both the nonproliferative and proliferative form, were at higher risk for nAMD when compared to diabetic patients without DR or normal controls [39]. Vascular endothelial growth factor (VEGF) seems to play an important role in both DR and AMD, and anti-VEGF treatment are useful for both [76], [77]. Apolipoproteins are also involved. Lower apoAI and higher apoB and apoB/AI levels, biomarkers for diabetic retinopathy [78], are involved in the pathogenesis of cardiovascular diseases [79], which is a risk factor for AMD [8], [59]. Meanwhile, mitochondrial dysfunctions have been reported to contribute to metabolic disorders as well as AMD [74], [80], [81]. All these suggested that hyperglycemia probably affects the function and structure of the retinal pigment epithelium, Bruch membrane, and the choroidal circulation [47], thus increase the risk of AMD. Our study indicates a potential relationship between diabetes and late AMD, but further evidences from more epidemiological and biological investigations are required.

To enhance the reliability of our results, we adopted quality assessment tools recommended by the AHRQ and NOS for observational studies. Only studies discussing diabetes as the main exposure or providing adjusted ORs/HRs/RRs were included in the present meta-analysis for a more precise association of diabetes as a relatively independent risk factor for AMD. In addition, for studies reporting duplicated cohorts, only those with the latest follow-up information or provides better adjusted results were included. Subgroup analysis was performed to affirm the association and to explorer the sources of the heterogeneity. Meanwhile, our study entailed some limitations. Data obtained from prospective cohort studies would be more convincing. But the number of prospective cohort studies was quite limited. Retrospective cohort, cross-sectional, and case-control studies were also included in the present study, which may partly help to reflect the association between diabetes and AMD. However, these studies have limitation. Retrospective cohort studies use healthcare databases and have inherent methodological limitations, which may obscure the association between diabetes and AMD [82]. Cross-sectional does not establish temporality and case-control studies may introduce selection bias and established temporality [82]. Early AMD can be further classified into more specific categories. Herein, we could only judge the relationship between diabetes and early AMD. Other than diabetic status, plenty of other risk factors have been suggested for AMD. Although we have tried to narrow down the influence of other risk factors by selecting studies with adjusted data, some included studies only reported data adjusted for age and sex, and the number of studies providing multivariate-adjusted data was quite limited. With the limited information provided by each individual study, therefore, this present meta-analysis only deals with the relationship between diabetic disease status and risk of AMD, but not the specific type of diabetes, the disease course, and blood glucose levels.

In conclusion, results of this meta-analysis indicate diabetes as a potential risk factor for AMD, especially for its late form. No clear association between diabetes and early AMD is identified. More longitudinal studies are needed to ascertain the association between diabetes and AMD. And biological studies involving the inflammatory pathways might help understand the molecular basis behind this association.

## Supporting Information

Table S1 **Quality Assessment for Included Cohort Studies.**
(DOCX)

Table S2 **Quality Assessment for Cross-Sectional Studies.**
(DOCX)

Table S3 **Quality Assessment for Case-Control Studies.**
(DOCX)

Checklist S1 (DOC)

Appendix S1 **Search Terms Used in the Present Study in Different Databases.**
(DOCX)
