# Supplementary material for: Diabetes Mellitus and Risk of Age-Related Macular Degeneration: A Systematic Review and Meta-Analysis
Source: PLoS One. 2014 Sep 19;9(9):e108196. doi: 10.1371/journal.pone.0108196 (PMC4169602; doi:10.1371/journal.pone.0108196)
Supplement: Table S1 — Quality Assessment for Included Cohort Studies. (DOCX) [file pone.0108196.s001.docx]

| **Table S1. Quality Assessment for Included Cohort Studies** | | | | | | | | | | | |
| --- | --- | --- | --- | --- | --- | --- | --- | --- | --- | --- | --- |
| **Author (Publication Year)** | **Quality Indicators From Newcastle-Ottawa Scale** | | | | | | | | | | |
|  | **Selection** | | | | | | **Comparability** | | **Outcome** | | |
|  | **1A** | **1B** | **2** | **3A** | **3B** | **4** | **5A** | **5B** | **6** | **7** | **8** |
| Tomany (2004) |  |  |  |  |  |  |  |  |  |  |  |
| *BDES* | Yes | Yes | Yes | Yes | No | Yes | Yes | Yes | Yes | Yes | Yes |
| *BMES* | Yes | Yes | Yes | Yes | No | Yes | Yes | Yes | Yes | Yes | Yes |
| *RS* | Yes | Yes | Yes | Yes | No | Yes | Yes | Yes | Yes | Yes | Yes |
| Leske (2006) | Yes | Yes | Yes | Yes | No | Yes | Yes | No | Yes | Yes | Yes |
| Yasuda (2009) | Yes | Yes | Yes | Yes | No | Yes | Yes | No | Yes | Yes | Yes |
| Shalev (2011) | No | Yes | No | Yes | No | Yes | Yes | Yes | Yes | Yes | Yes |
| Hahn (2013) | No | Yes | No | Yes | No | Yes | Yes | Yes | Yes | Yes | Yes |
| **Abbreviations:** 1A: Truly representative of the community; 1B: Show somewhat representative of the community; 2: The non exposed cohort is drawn from the same community as the exposed cohort; 3A: Ascertainment of exposure is through secure record; 3B: Ascertainment of exposure is through structured interview; 4: Outcome of interest is not presented in the beginning of the study; 5A: Study controls for the most important factor; 5B: Study controls for any additional factor; 6: Assessment of outcomes is independent blind or record linkage; 7: Follow-up is long enough for outcomes to occur; 8: Complete follow-up or subjects lost to follow up are unlikely to introduce bias. BDES: Beaver Dam Eye Study; BMES: Blue Mountains Eye Study; RS: Rotterdam Study. | | | | | | | | | | | |
