# Supplementary material for: Diabetes Mellitus and Risk of Age-Related Macular Degeneration: A Systematic Review and Meta-Analysis
Source: PLoS One. 2014 Sep 19;9(9):e108196. doi: 10.1371/journal.pone.0108196 (PMC4169602; doi:10.1371/journal.pone.0108196)
Supplement: Table S2 — Quality Assessment for Cross-Sectional Studies. (DOCX) [file pone.0108196.s002.docx]

| **Table S2. Quality Assessment for Cross-Sectional Studies** | | | | | | | | | | | |
| --- | --- | --- | --- | --- | --- | --- | --- | --- | --- | --- | --- |
| **Author (Publication Year)** | **Quality Indicators From AHRQ** | | | | | | | | | | |
|  | **1** | **2** | **3** | **4** | **5** | **6** | **7** | **8** | **9** | **10** | **11** |
| Delcourt (2001) | Yes | Yes | Yes | Yes | No | Yes | Yes | Yes | Yes | Yes | Unclear |
| Vaičaitienė (2003) | Yes | Yes | Yes | Yes | No | No | No | No | No | No | Unclear |
| Duan (2007) | Yes | Yes | Yes | Yes | No | Yes | Yes | Yes | Yes | Yes | Yes |
| Klein (2007) | Yes | Yes | Yes | Yes | No | Yes | Yes | Yes | No | Yes | Unclear |
| Topouzis (2009) | Yes | Yes | Yes | Yes | No | Yes | Yes | Yes | Yes | Yes | Unclear |
| Xu (2009) | Yes | Yes | Yes | Yes | No | Yes | No | No | No | Yes | Yes |
| Choi (2011) | Yes | Yes | Yes | Yes | No | Yes | Yes | Yes | No | Yes | Unclear |
| Cheung (2013) |  |  |  |  |  |  |  |  |  |  |  |
| *SIES* | Yes | Yes | Yes | Yes | No | Yes | Yes | Yes | Yes | Yes | Unclear |
| *CIEMS* | Yes | Yes | Yes | Yes | No | Yes | Yes | Yes | Yes | Yes | Unclear |
| **Abbreviations:** 1. Define the source of information (survey, record review); 2. List inclusion and exclusion criteria for exposed and unexposed subjects (cases and controls) or refer to previous publications; 3. Indicate time period used for identifying patients; 4. Indicate whether or not subjects were consecutive if not population-based; 5. Indicate if evaluators of subjective components of study were masked to other aspects of the status of the participants; 6. Describe any assessments undertaken for quality assurance purposes; 7. Explain any patient exclusions from analysis; 8. Describe how confounding was assessed and/or controlled; 9. If applicable, explain how missing data were handled in the analysis; 10. Summarize patient response rates and completeness of data collection; 11. Clarify what follow-up, if any, was expected and the percentage of patients for which incomplete data or follow-up was obtained AREDS: Age-Related Eye Disease Study; SIES: Singapore Indian Eye Study; CIEMS: Central India Eye and Medical Study. | | | | | | | | | | | |
