# Supplementary material for: Diabetes Mellitus and Risk of Age-Related Macular Degeneration: A Systematic Review and Meta-Analysis
Source: PLoS One. 2014 Sep 19;9(9):e108196. doi: 10.1371/journal.pone.0108196 (PMC4169602; doi:10.1371/journal.pone.0108196)
Supplement: Table S3 — Quality Assessment for Case-Control Studies. (DOCX) [file pone.0108196.s003.docx]

| **Table S3. Quality Assessment for Case-Control Studies** | | | | | | | | | | | |
| --- | --- | --- | --- | --- | --- | --- | --- | --- | --- | --- | --- |
| **Author (Publication Year)** | **Quality Indicators From Newcastle-Ottawa Scale** | | | | | | | | | | |
|  | **Selection** | | | |  | **Comparability** | |  | **Exposure** | | |
|  | **1** | **2** | **3** | **4** |  | **5A** | **5B** |  | **6** | **7** | **8** |
| Blumenkranz (1986) | Yes | Yes | No | Yes |  | Yes | Yes |  | Yes | Yes | No |
| Ross (1998) | Yes | Yes | No | Yes |  | Yes | No |  | Yes | Yes | No |
| McGwin Jr (2003) | Yes | Yes | No | Yes |  | Yes | No |  | Yes | Yes | No |
| Moeini (2005) | Yes | Yes | No | Yes |  | Yes | Yes |  | Yes | Yes | No |
| Alexander (2007) | Yes | Yes | No | Yes |  | Yes | Yes |  | Yes | Yes | No |
| Kim (2008) | Yes | Yes | Yes | Yes |  | Yes | Yes |  | Yes | Yes | No |
| Lin (2008) | Yes | Yes | No | Yes |  | Yes | Yes |  | Yes | Yes | No |
| Nitsch (2008) | Yes | Yes | No | Yes |  | Yes | Yes |  | Yes | Yes | No |
| Cackett (2011) | Yes | Yes | No | Yes |  | Yes | Yes |  | Yes | Yes | No |
| Sogut (2013) | Yes | Yes | No | Yes |  | Yes | Yes |  | Yes | Yes | No |
| Torre (2013) | Yes | Yes | No | Yes |  | Yes | Yes |  | Yes | Yes | No |
| **Abbreviations:** 1: Case is defined with independent validations; 2: Consecutive or obvious representative series of cases; 3: Community controls; 4: Controls are defined as no history of disease; 5A: Study controls for the most important factor; 5B: Study controls for any additional factor; 6: Ascertainment of exposure is secure record; 7: Same method of ascertainment for cases and controls; 8: Same non-response rates for both groups. | | | | | | | | | | | |
