# Supplementary material for: Diabetes Mellitus and Risk of Age-Related Macular Degeneration: A Systematic Review and Meta-Analysis
Source: PLoS One. 2014 Sep 19;9(9):e108196. doi: 10.1371/journal.pone.0108196 (PMC4169602; doi:10.1371/journal.pone.0108196)
Supplement: Appendix S1 — Search Terms Used in the Present Study in Different Databases. (DOCX) [file pone.0108196.s005.docx]

**Appendix S1. Search Terms Used in the Present Study in Different Databases**

MEDLINE, EMBASE (OVID), and The Cochrane Library

1. exp Macular Degeneration/
2. exp Retinal Degeneration/
3. ((macul* or retina* or choroid*) adj4 degener*).tw.
4. maculopath*.tw.
5. (macul* adj3 dystroph*).tw.
6. ((macul* or geographic) adj2 atroph*).tw.
7. 1 or 2 or 3 or 4 or 5 or 6
8. exp diabetes mellitus/
9. (diabete* or diabetic).ti,ab.
10. 8 or 9
11. (prevalence* or incidence* or occurrence or risk* or protecti*).mp.
12. ((case adj1 control) or (cross adj1 section*)).mp.
13. exp epidemiology/
14. 11 or 12 or 13
15. 7 and 10 and 14
